# Association of *ALDH2* rs671 and *MTHFR* rs1801133 polymorphisms with hypertension among Hakka people in Southern China

**DOI:** 10.1186/s12872-022-02577-x

**Published:** 2022-03-27

**Authors:** Heming Wu, Qingyan Huang, Zhikang Yu, Zhixiong Zhong

**Affiliations:** 1 Center for Precision Medicine, Meizhou People’s Hospital (Huangtang Hospital), Meizhou Academy of Medical Sciences, Meizhou, People’s Republic of China; 2 Guangdong Provincial Key Laboratory of Precision Medicine and Clinical Translational Research of Hakka Population, Meizhou People’s Hospital (Huangtang Hospital), Meizhou Academy of Medical Sciences, Meizhou, People’s Republic of China; 3 Guangdong Provincial Engineering and Technology Research Center for Clinical Molecular Diagnostics and Antibody Therapeutics, Meizhou People’s Hospital (Huangtang Hospital), Meizhou Academy of Medical Sciences, Meizhou, People’s Republic of China

**Keywords:** Hypertension, Acetaldehyde dehydrogenase 2, Methylenetetrahydrofolate reductase, Polymorphism, Hakka

## Abstract

**Background:**

Genetic factors play an important role in susceptibility to hypertension. Herein, the association between acetaldehyde dehydrogenase 2 (ALDH2) and methylenetetrahydrofolate reductase (MTHFR) gene polymorphisms and hypertension was analyzed among Hakka population in southern China.

**Methods:**

A total of 3057 hypertensive patients and 2215 controls were enrolled. The *ALDH2* rs671 and *MTHFR* rs1801133 genotyping were analyzed using gene chip. Relevant information and medical records of these subjects were collected.

**Results:**

Hypertensive patients with *ALDH2* rs671 G/A heterozygous had lower systolic blood pressure (SBP) than other genotypes (*P* < 0.001), while hypertensive patients with A allele had lower diastolic blood pressure (DBP) than patients with G allele (*P* < 0.001). The level of plasma homocysteine (Hcy) in patients with *MTHFR* CC, CT and TT genotypes showed an increasing trend (*P* < 0.001). The *ALDH2* G/A genotype in the co-dominant model (adjusted OR 1.251, 95% CI 1.024–1.528, *P* = 0.028) and *ALDH2* A/A genotype in the recessive model (adjusted OR 1.221, 95% CI 1.008–1.478, *P* = 0.041) were significant risk factors for the presence of hypertension. The *MTHFR* C/T genotype in the co-dominant model (adjusted OR 1.307, 95% CI 1.039–1.643, *P* = 0.022) and *MTHFR* C/T and T/T genotypes in the dominant model (adjusted OR 1.281, 95% CI 1.146–1.430, *P* < 0.001) were significant risk factors for the presence of hypertension. Further, logistic regression analysis showed that age, smoking, alcohol consumption, hyperhomocysteinemia, and high level of serum TG, Apo-A1, Apo-B were significant risks for hypertension.

**Conclusions:**

In summary, *ALDH2* rs671 G/A, A/A genotypes and *MTHFR* rs1801133 C/T, T/T genotypes may be risk factors for hypertension in this Chinese Hakka population.

## Introduction

Hypertension is one of the common chronic diseases at present, and it is characterized by elevated systemic arterial pressure. It can be accompanied with clinical syndromes of functional or organic damaging of organs such as heart, brain, and kidney [1]. Over the past decade, hypertension has become the leading cause of the global burden of diseases [2]. Hypertension is defined as systolic blood pressure (SBP) ≥ 140 mm Hg and/or diastolic blood pressure (DBP) ≥ 90 mm Hg [3–5]. In 1975, the number of people with hypertension worldwide was 590 million (prevalence was 14.5%), it had increased to 1.13 billion (prevalence was 15.3%) in 2015, and it is predicted that the number of people with hypertension worldwide will continue to increase to 1.56 billion in 2025 [2]. The prevalence of hypertension is on the rise.

The prevention and control of hypertension are important public health problems in China. A survey completed in 2017 showed that among 1,738,886 middle-aged and elderly people from 31 provinces on the mainland of China, the overall prevalence rate was 45%, the diagnostic rate was 45%, the treatment rate was 30%, and the control rate was 7% [6]. In 2018, the prevalence rate of hypertension in adults in China was 27.5%. Among the hypertensive persons, 41.0% of the patients were aware of their blood pressure status, 34.9% of the patients were taking antihypertensive medicines, and 11.0% of the patients had their blood pressure controlled [7]. Given the higher prevalence rate of hypertension and lower blood pressure measurement rate in Chinese adults, as well as grim status of awareness, treatment and control of hypertension in patients, more efforts should be made in prevention and control of hypertension, such as improved risk factor intervention and case management.

Risk factors for hypertension include genetic factors, age and a variety of adverse lifestyle [8]. Worldwide studies have demonstrated that the genetic tendency of hypertension is very obvious, with 30% ~ 50% of an individual's risk of hypertension attributable to genetic factors [9]. People with a family history of hypertension have higher lifetime risk of hypertension than those without a family history of hypertension [10]. With the development of molecular biology technology, many studies were devoted to the genetic etiology of hypertension, and remain to be elucidated.

Acetaldehyde dehydrogenase 2 (ALDH2) is a key enzyme involved in alcohol metabolism [11]. In addition, it is a protective factor against oxidative stress, so deficiency in ALDH2 increases oxidative stress in the body [12]. Oxidative stress significantly contributes to the vascular dysfunction and renal damage associated with hypertension [13, 14]. Animal experiments have shown that decreased ALDH2 expression was associated with progression of hypertension [15]. Activation of ALDH2 improves coronary angiogenesis to ameliorate cardiometabolic diseases [16]. ALDH2 activity is related to the occurrence and development of hypertension. The ALDH2 activity in vivo is closely related to *ALDH2* polymorphisms. *ALDH2* gene is located on chromosome 12q24.2 and some polymorphisms have been found. The most common polymorphism is Glu504Lys polymorphism (SNP rs671, G1510A), leading to the decreased ALDH2 activity [17].

There are evidences that high plasma homocysteine (Hcy) level is a risk factor for cardiovascular and cerebrovascular diseases. High Hcy level has a synergistic effect with hypertension and can significantly increase the risk of vascular diseases [18, 19]. Methylenetetrahydrofolate reductase (MTHFR) is a key enzyme in the process of effecting the metabolism of Hcy [20]. Homeostasis around vascular endothelium is a function of the equilibrium between the bioavailability of nitric oxide (NO) and oxidizing reactive oxygen species (ROS). NO plays a role in enhancing vasodilatation and reducing platelet aggression and adhesion in vascular endothelium, and plays an important role in hypertension. Levels of Hcy and MTHFR, play a determining role in circulating levels of NO [21]. The MTHFR activity in vivo is closely related to the *MTHFR* gene polymorphisms. The human *MTHFR* gene is located on chromosome 1p36.22 and composed of 12 exons [22]. *MTHFR* C677T (SNP rs1801133, Ala222Val) is the most common polymorphism of *MTHFR* gene, and the mutant allele was associated with high level of Hcy [23].

We hypothesized that polymorphisms of the *ALDH2* and *MTHFR* gene that result in reduced enzyme activity may increase the risk of hypertension. The results of studies on the relationship between *ALDH2*, *MTHFR* gene polymorphisms and hypertension are inconsistent. Several limitations of the previous studies should be acknowledged such as most studies have focused on individual local populations and only focused on the relationship between a single gene and hypertension, ignoring gene-to-gene interactions. In the present study, the association of *ALDH2* rs671 and *MTHFR* rs1801133 polymorphisms with hypertension was analyzed among Hakka people in Southern China.

## Materials and methods

### Data collection

The study included 3057 hypertensive patients and 2215 controls. Data of the participants (including age, sex, smoking history, alcohol consumption, medical history, and serum lipid levels) were collected from hospital information system (HIS) and laboratory information system (LIS) of Meizhou People’s Hospital (Huangtang Hospital), Guangdong province, China, from April 2016 to December 2020. Inclusive criteria: (1) Patients with clinically diagnosed hypertension. (2) Age ≥ 30 years old. (3) The participants are Hakka people based on questionnaires about the ethnicity. Exclusion criteria: (1) Age < 30 years old. (2) Participants with secondary hypertension. (3) Participants with severe liver and kidney failure, and serious infections. All control subjects were randomly selected from the Physical Examination Center of the Meizhou People’s Hospital during the same period. This retrospective case control study was performed in accordance with the Declaration of Helsinki and approved by the Human Ethics Committees of Meizhou People's Hospital.

### Sample collection, DNA extraction and genotyping

Venous blood was collected from each subject. Genomic DNA was extracted from whole blood using a QIAamp DNA Blood Mini Kit (Qiagen GmbH, North Rhine-Westphalia, Germany) according to the protocol. Polymerase chain reaction (PCR)-gene chip method was used for *ALDH2* and *MTHFR* genotyping (BaiO Technology Co, Ltd, Shanghai, China). PCR was used to amplify the target fragments: initial denaturation for 5 min at 94 °C, followed by 35 cycles of denaturation for 25 s at 94 °C, annealing for 25 s at 56 °C and chain elongation for 25 s at 72 °C. The specific hybridization reaction was carried out between the amplification products and the wild-type or mutation-type probes fixed on the chip, the genotype of the sample to be detected was determined according to the color reaction of the specific hybridization signal.

### Statistical analysis

Data analysis was performed using SPSS statistical software version 21.0 (IBM Inc., USA). Student’s t-test or the Mann–Whitney U test was used for continuous data analysis. Genotype composition ratios and allele frequencies of groups were analyzed by the Chi-square test. Logistic regression analysis was applied to examine the relationship between *ALDH2* and *MTHFR* polymorphisms and various factors in hypertension. *P* < 0.05 was considered statistically significant.

## Results

### Population characteristics

A total of 3057 hypertensive patients (2040 men and 1017 women) and 2215 controls (1502 men and 713 women) were enrolled. The hypertensive patients’ average age was 67.32 ± 11.78 years, with 65.32 ± 12.65 years for controls. There were statistically significant differences in the percentage of smokers (*P* < 0.001), percentage of alcoholism (*P* < 0.001), and prevalence of diabetes (*P* < 0.001). The Hcy level (*P* < 0.001), serum lipid levels such as triglycerides (TG) (*P* = 0.016), total cholesterol (TC) (*P* = 0.001), low-density lipoprotein-cholesterol (LDL-C) (*P* = 0.003), apolipoprotein A1 (Apo-A1) (*P* < 0.001), and apolipoprotein B (Apo-B) (*P* < 0.001), were also significantly higher in the hypertension group compared with controls, except for high-density lipoprotein-cholesterol (HDL-C) (*P* = 0.109) (Table 1).

**Table 1:** Clinical characteristics of hypertensive patients and control participants

	Total (n = 5272)	Hypertensive patients (n = 3057)	Controls (n = 2215)	*P* values
Age, years	66.48 ± 12.19	67.32 ± 11.78	65.32 ± 12.65	**< 0.001**
Gender				
Male, n (%)	3542 (67.19%)	2040 (66.73%)	1502 (67.81%)	0.422
Female, n (%)	1730 (32.81%)	1017 (33.27%)	713 (32.19%)	
Smokers, n (%)	1509 (28.62%)	799 (26.14%)	710 (32.05%)	**< 0.001**
Alcoholism, n (%)	349 (6.62%)	157 (5.14%)	192 (8.67%)	**< 0.001**
Diabetes, n (%)	1356 (25.72%)	950 (31.08%)	406 (18.33%)	**< 0.001**
SBP, mmHg	142.53 ± 32.94	152.42 ± 35.55	128.89 ± 22.74	**< 0.001**
DBP, mmHg	82.83 ± 15.62	86.99 ± 15.46	77.09 ± 13.91	**< 0.001**
Hcy, μmol/L	16.66 ± 8.10	17.19 ± 8.12	15.93 ± 8.02	**< 0.001**
TG, mmol/L	1.77 ± 1.68	1.81 ± 1.57	1.70 ± 1.82	**0.016**
TC, mmol/L	4.88 ± 1.39	4.93 ± 1.31	4.80 ± 1.48	**0.001**
HDL-C, mmol/L	1.26 ± 0.39	1.27 ± 0.38	1.25 ± 0.41	0.109
LDL-C, mmol/L	2.73 ± 0.97	2.76 ± 0.93	2.68 ± 1.03	**0.003**
Apo-A1, g/L	1.10 ± 0.33	1.12 ± 0.31	1.08 ± 0.35	**< 0.001**
Apo-B, g/L	0.85 ± 0.29	0.86 ± 0.28	0.83 ± 0.30	**< 0.001**

The bold values indicate *P* < 0.05, with statistically significant difference

Values for age expressed as mean ± SD. SBP, systolic blood pressure; DBP, diastolic blood pressure; Hcy, homocysteine; TG, triglycerides; TC, total cholesterol; HDL-C, high-density lipoprotein-cholesterol; LDL-C, low-density lipoprotein-cholesterol; Apo-A1, apolipoprotein A1; Apo-B, apolipoprotein B

### Frequencies of *ALDH2* rs671, *MTHFR* rs1801133 genotypes and alleles in hypertensive patients and controls

Frequencies of *ALDH2* rs671, *MTHFR* rs1801133 genotypes and alleles in hypertensive patients and controls are shown in Table 2. The genotype distributions of *ALDH2* and *MTHFR* genes in both the hypertensive patients (χ^2^ = 3.253, *P* = 0.197 and χ^2^ = 2.770, *P* = 0.250) and controls (χ^2^ = 0.174, *P* = 0.917 and χ^2^ = 0.026, *P* = 0.987) were consistent with Hardy–Weinberg equilibrium, respectively. In hypertensive patients, the frequency of the *ALDH2* G/G, G/A, and A/A genotype was 47.17%, 44.42%, and 8.41%, respectively, with the frequency of the *ALDH2* G and A allele was 69.38% and 30.62%, respectively. In controls, the frequency of the *ALDH2* G/G, G/A, and A/A genotype was 46.46%, 43.79%, and 9.75%, respectively, with the frequency of the G and A allele was 68.35% and 31.65%, respectively. There were no significant differences in *ALDH2* genotype and allele distribution between hypertensive patients and controls (all *P* > 0.05).

**Table 2:** Frequencies of *ALDH2* rs671, *MTHFR* rs1801133 genotypes and alleles in hypertensive patients and controls

Genotype/allele	Hypertensive patients (n = 3057)	Controls (n = 2215)	χ^2^	*P* value
*ALDH2* rs671				
G/G	1442 (47.17%)	1029 (46.46%)	2.844	0.242
G/A	1358 (44.42%)	970 (43.79%)		
A/A	257 (8.41%)	216 (9.75%)		
G	4242 (69.38%)	3028 (68.35%)	1.272	0.259
A	1872 (30.62%)	1402 (31.65%)		
HWE (χ^2^, *P*)	χ^2^ = 3.253, *P* = 0.197	χ^2^ = 0.174, *P* = 0.917		
*MTHFR* rs1801133				
C/C	1555 (50.87%)	1264 (57.07%)	19.842	**< 0.001**
C/T	1287 (42.10%)	816 (36.84%)		
T/T	215 (7.03%)	135 (6.09%)		
C	4397 (71.92%)	3344 (75.49%)	16.760	**< 0.001**
T	1717 (28.08%)	1086 (24.51%)		
HWE (χ^2^, *P*)	χ^2^ = 2.770, *P* = 0.250	χ^2^ = 0.026, *P* = 0.987		

The bold values indicate *P* < 0.05, with statistically significant difference

HWE, Hardy Weinberg Equilibrium

In hypertensive patients, the frequency of the *MTHFR* C/C, C/T, and T/T genotype was 50.87%, 42.10%, and 7.03%, respectively, with the frequency of the *MTHFR* C and T allele was 71.92% and 28.08%, respectively. In controls, the frequency of the *MTHFR* C/C, C/T, and T/T genotype was 57.07%, 36.84%, and 6.09%, respectively, with the frequency of the C and T allele was 75.49% and 24.51%, respectively. The results showed that there were significant differences in *MTHFR* genotype and allele distribution between hypertensive patients and controls (all *P* < 0.001) (Table 2).

### Clinical characteristics of hypertensive patients stratified by *ALDH2* and *MTHFR* variants

While most laboratory test results were compared among hypertensive patients stratified by *ALDH2* rs671 genotypes and alleles, hypertensive patients with G/A heterozygous had lower SBP than other genotypes (149.42 ± 25.26 mmHg vs. 154.94 ± 24.30 mmHg in G/G genotype and 154.07 ± 91.08 mmHg in A/A genotype, *P* < 0.001), while hypertensive patients with A allele (150.16 ± 43.07 mmHg) had lower SBP than patients with G allele (152.27 ± 24.92 mmHg) (*P* = 0.040). There were significant differences in DBP of hypertensive patients among *ALDH2* genotypes (88.84 ± 15.36, 85.52 ± 15.20, 84.38 ± 16.25 mmHg in G/G, G/A, and A/A genotypes, respectively, *P* < 0.001), while hypertensive patients with A allele (85.34 ± 15.37 mmHg) had lower DBP than patients with G allele (87.23 ± 15.37 mmHg) (*P* < 0.001). Hypertensive patients with G/A heterozygous had lower LDL-C level than other genotypes (2.70 ± 0.92 mmol/L vs. 2.80 ± 0.92 mmol/L in G/G genotype and 2.87 ± 0.98 mmol/L in A/A genotype, *P* = 0.003). There were no differences in other laboratory indicators among the different *ALDH2* genotypes and alleles (Table 3).

**Table 3:** Clinical characteristics of hypertensive patients stratified by *ALDH2* rs671 genotypes and alleles

Clinical characteristics	G/G (n = 1442)	G/A (n = 1358)	A/A (n = 257)	*P* values	G allele (G/G + G/A) (n = 2800)	A allele (G/A + A/A) (n = 1615)	*P* values
Age, years	66.62 ± 11.98	67.79 ± 11.55	68.79 ± 11.66	**0.004**	67.18 ± 11.78	67.95 ± 11.57	**0.037**
Gender							
Male, n (%)	1041 (72.19%)	834 (61.41%)	165 (64.20%)	**< 0.001**	1875 (66.96%)	999 (61.86%)	**0.001**
Female, n (%)	401 (27.81%)	524 (38.59%)	92 (35.80%)		925 (33.04%)	616 (38.14%)	
Smokers, n (%)	422 (29.26%)	322 (23.71%)	55 (21.40%)	**0.001**	744 (26.57%)	377 (23.34%)	**0.018**
Alcoholism, n (%)	104 (7.21%)	49 (3.61%)	4 (1.56%)	**< 0.001**	153 (5.46%)	53 (3.28%)	**0.001**
Diabetes, n (%)	432 (29.96%)	442 (32.55%)	76 (29.57%)	0.289	874 (31.21%)	518 (32.07%)	0.568
SBP, mmHg	154.94 ± 24.30	149.42 ± 25.26	154.07 ± 91.08	**< 0.001**	152.27 ± 24.92	150.16 ± 43.07	**0.040**
DBP, mmHg	88.84 ± 15.36	85.52 ± 15.20	84.38 ± 16.25	**< 0.001**	87.23 ± 15.37	85.34 ± 15.37	**< 0.001**
Hcy, μmol/L	17.52 ± 8.83	16.81 ± 7.19	17.29 ± 8.57	0.066	17.18 ± 8.08	16.89 ± 7.42	0.236
TG, mmol/L	1.82 ± 1.67	1.80 ± 1.43	1.85 ± 1.65	0.889	1.81 ± 1.56	1.81 ± 1.47	0.997
TC, mmol/L	4.97 ± 1.32	4.87 ± 1.30	5.03 ± 1.32	0.062	4.92 ± 1.31	4.90 ± 1.30	0.531
HDL-C, mmol/L	1.28 ± 0.39	1.26 ± 0.38	1.25 ± 0.35	0.243	1.27 ± 0.38	1.26 ± 0.37	0.296
LDL-C, mmol/L	2.80 ± 0.92	2.70 ± 0.92	2.87 ± 0.98	**0.003**	2.75 ± 0.92	2.73 ± 0.93	0.457
Apo-A1, g/L	1.12 ± 0.31	1.11 ± 0.31	1.10 ± 0.32	0.545	1.12 ± 0.31	1.11 ± 0.31	0.496
Apo-B, g/L	0.87 ± 0.28	0.84 ± 0.27	0.89 ± 0.30	**0.006**	0.86 ± 0.27	0.85 ± 0.28	0.376

The bold values indicate *P* < 0.05, with statistically significant difference

The levels of Hcy in patients with *MTHFR* CC, CT and TT genotype showed an increasing trend (16.31 ± 6.34 μmol/L, 17.22 ± 7.63 μmol/L, 23.32 ± 16.03 μmol/L) (*P* < 0.001), while hypertensive patients with T allele (18.09 ± 9.54 μmol/L) had higher Hcy level than patients with C allele (16.72 ± 6.96 μmol/L) (*P* < 0.001). There were no differences in other laboratory indicators among the different the *MTHFR* genotypes and alleles (Table 4).

**Table 4:** Clinical characteristics of hypertensive patients stratified by *MTHFR* rs1801133 genotypes and alleles

Clinical characteristics	C/C (n = 1555)	C/T (n = 1287)	T/T (n = 215)	*P* values	C allele (C/C + C/T) (n = 2842)	T allele (C/T + T/T) (n = 1502)	*P* values
Age, years	67.26 ± 11.87	67.63 ± 11.63	65.90 ± 11.96	0.130	67.43 ± 11.76	67.38 ± 11.69	0.908
Gender							
Male, n (%)	1087 (69.90%)	812 (63.09%)	141 (65.58%)	**0.001**	1899 (66.82%)	953 (63.45%)	**0.027**
Female, n (%)	468 (30.10%)	475 (36.91%)	74 (34.42%)		943 (33.18%)	549 (36.55%)	
Smokers, n (%)	438 (28.17%)	308 (23.93%)	53 (24.65%)	**0.033**	746 (26.25%)	361 (24.03%)	0.111
Alcoholism, n (%)	76 (4.89%)	69 (5.36%)	12 (5.58%)	0.811	145 (5.10%)	81 (5.39%)	0.681
Diabetes, n (%)	497 (31.96%)	396 (30.77%)	57 (26.51%)	0.257	893 (31.42%)	453 (30.16%)	0.392
SBP, mmHg	152.90 ± 25.40	152.38 ± 46.09	149.16 ± 24.17	0.351	152.66 ± 36.26	151.92 ± 43.64	0.548
DBP, mmHg	87.10 ± 15.49	86.92 ± 15.50	86.59 ± 15.08	0.881	87.02 ± 15.49	86.87 ± 15.44	0.765
Hcy, μmol/L	16.31 ± 6.34	17.22 ± 7.63	23.32 ± 16.03	**< 0.001**	16.72 ± 6.96	18.09 ± 9.54	**< 0.001**
TG, mmol/L	1.81 ± 1.65	1.82 ± 1.44	1.81 ± 1.69	0.999	1.81 ± 1.56	1.82 ± 1.48	0.982
TC, mmol/L	4.95 ± 1.31	4.90 ± 1.32	4.95 ± 1.25	0.574	4.93 ± 1.32	4.91 ± 1.31	0.620
HDL-C, mmol/L	1.27 ± 0.39	1.26 ± 0.37	1.27 ± 0.34	0.858	1.27 ± 0.38	1.26 ± 0.37	0.738
LDL-C, mmol/L	2.78 ± 0.91	2.74 ± 0.94	2.79 ± 0.93	0.568	2.76 ± 0.93	2.75 ± 0.94	0.691
Apo-A1, g/L	1.12 ± 0.31	1.12 ± 0.32	1.13 ± 0.29	0.888	1.12 ± 0.32	1.12 ± 0.31	0.898
Apo-B, g/L	0.86 ± 0.27	0.86 ± 0.28	0.88 ± 0.27	0.644	0.86 ± 0.28	0.86 ± 0.28	0.888

The bold values indicate *P* < 0.05, with statistically significant difference

### Association of *ALDH2* rs671 and *MTHFR* rs1801133 polymorphisms with hypertension

The possible association of the *ALDH2* genotypes with potential risk factors for hypertension were based on three genetic modes of inheritance, such as the co-dominant mode (*ALDH2* G/A vs. *ALDH2* G/G, *ALDH2* A/A vs. *ALDH2* G/G), dominant mode (*ALDH2* G/A plus *ALDH2* A/A vs. *ALDH2* G/G), and recessive mode (*ALDH2* A/A vs. *ALDH2* G/G plus *ALDH2* G/A) models. The *ALDH2* G/A genotype in the co-dominant model (*ALDH2* G/A vs. *ALDH2* G/G) (age-, smoking-, and drinking-adjusted OR 1.251, 95% CI 1.024–1.528, *P* = 0.028) and *ALDH2* A/A genotype in the recessive model (*ALDH2* A/A vs. *ALDH2* G/G plus *ALDH2* G/A) (age-, smoking-, and drinking-adjusted OR 1.221, 95% CI 1.008–1.478, *P* = 0.041) were significant risk factors for the presence of hypertension.

The possible association of the *MTHFR* genotypes with potential risk factors for hypertension were based on three genetic modes of inheritance, such as the co-dominant mode (*MTHFR* C/T vs. *MTHFR* C/C, *MTHFR* T/T vs. *MTHFR* C/C), dominant mode (*MTHFR* C/T plus *MTHFR* T/T vs. *MTHFR* C/C), and recessive mode (*MTHFR* T/T vs. *MTHFR* C/C plus *MTHFR* C/T) models. The *MTHFR* C/T genotype in the co-dominant model (*MTHFR* C/T vs. *MTHFR* C/C) (age-, smoking-, and drinking-adjusted OR 1.307, 95% CI 1.039–1.643, *P* = 0.022) and *MTHFR* C/T and T/T genotypes in the dominant model (*MTHFR* C/T plus *MTHFR* T/T vs. *MTHFR* C/C) (age-, smoking-, and drinking-adjusted OR 1.281, 95% CI 1.146–1.430, *P* < 0.001) were significant risk factors for the presence of hypertension (Table 5).

**Table 5:** Association of *ALDH2* rs671 and *MTHFR* rs1801133 polymorphisms with hypertension

SNP	Model	Genotype	Hypertension	Control	Univariate OR (95% CI)	*P* values	Multivariate OR (95% CI)	*P* values*
*ALDH2* rs671	Co-dominant	G/G	1442 (47.17%)	1029 (46.46%)	1.000 (reference)			
		G/A	1358 (44.42%)	970 (43.79%)	1.178 (0.966–1.435)	0.105	1.251 (1.024–1.528)	**0.028**
		A/A	257 (8.41%)	216 (9.75%)	1.177 (0.965–1.435)	0.109	1.191 (0.975–1.455)	0.087
	Dominant	G/G	1442 (47.17%)	1029 (46.46%)	1.000 (reference)			
		G/A + A/A	1615 (52.83%)	1186 (53.54%)	1.029 (0.922–1.148)	0.608	1.082 (0.968–1.209)	0.166
	Recessive	G/G + G/A	2800 (91.59%)	1999 (90.25%)	1.000 (reference)			
		A/A	257 (8.41%)	216 (9.75%)	1.177 (0.974–1.423)	0.092	1.221 (1.008–1.478)	**0.041**
*MTHFR* rs1801133	Co-dominant	C/C	1555 (50.87%)	1264 (57.07%)	1.000 (reference)			
		C/T	1287 (42.10%)	816 (36.84%)	1.295 (1.031–1.626)	**0.026**	1.307 (1.039–1.643)	**0.022**
		T/T	215 (7.03%)	135 (6.09%)	1.010 (0.800–1.274)	0.935	1.024 (0.810–1.294)	0.844
	Dominant	C/C	1555 (50.87%)	1264 (57.07%)	1.000 (reference)			
		C/T + T/T	1502 (49.13%)	951 (42.93%)	1.284 (1.150–1.433)	**< 0.001**	1.281 (1.146–1.430)	**< 0.001**
	Recessive	C/C + C/T	2842 (92.97%)	2080 (93.91%)	1.000 (reference)			
		T/T	215 (7.03%)	135 (6.09%)	1.166 (0.933–1.456)	0.177	1.179 (0.942–1.475)	0.150

The bold values indicate *P* < 0.05, with statistically significant difference

*P values are adjusted for age, smoking, and drinking and estimated by logistic regression

### Association of other risk factors with hypertension

It is important to examine the association of traditional risk factors with hypertension because hypertension is associated with various factors, such as age, gender, cigarette smoking, alcohol consumption, and serum lipid level. The results of logistic regression analysis showed that age (adjusted OR 1.013, 95% CI 1.008–1.018, *P* < 0.001), smoking (adjusted OR 1.207, 95% CI 1.061–1.373, *P* = 0.004), alcohol consumption (adjusted OR 1.565, 95% CI 1.238–1.977, *P* < 0.001), hyperhomocysteinemia (adjusted OR 1.022, 95% CI 1.014–1.030, *P* < 0.001), and high level of serum TG (adjusted OR 1.061, 95% CI 1.008–1.117, *P* = 0.023), Apo-A1 (adjusted OR 1.870, 95% CI 1.428–2.449, *P* < 0.001), Apo-B (adjusted OR 1.598, 95% CI 1.047–2.440, *P* = 0.030) were significant risks for the presence of hypertension. However, high level of serum TC, LDL-C and low level of HDL-C were not significant risk factors (Table 6).

**Table 6:** Association of other risk factors with hypertension

Variables	Unadjusted values			Adjusted values		
	*P* value	OR	95% CI	*P* value	Adjusted OR	95% CI
Age	**< 0.001**	1.014	1.009–1.018	**< 0.001**	1.013	1.008–1.018
Smoking	**< 0.001**	1.333	1.182–1.504	**0.004**	1.207	1.061–1.373
Alcoholism	**< 0.001**	1.753	1.409–2.181	**< 0.001**	1.565	1.238–1.977
Hcy	**< 0.001**	1.021	1.014–1.029	**< 0.001**	1.022	1.014–1.030
TG	**0.018**	1.043	1.007–1.080	**0.023**	1.061	1.008–1.117
TC	**< 0.001**	1.074	1.032–1.118	0.398	0.937	0.805–1.090
HDL-C	0.105	1.122	0.976–1.290	0.163	0.828	0.635–1.079
LDL-C	**0.002**	1.092	1.032–1.156	0.909	1.011	0.833–1.228
Apo-A1	**< 0.001**	1.455	1.228–1.724	**< 0.001**	1.870	1.428–2.449
Apo-B	**< 0.001**	1.448	1.193–1.757	**0.030**	1.598	1.047–2.440

The bold values indicate *P* < 0.05, with statistically significant difference

OR, odds ratio; CI, confidence interval

## Discussion

Hypertension is a high risk factor of cardiovascular and cerebrovascular diseases in middle-aged and elderly people. Long-term hypertension will induce diabetes, heart failure, kidney disease, coronary artery disease, stroke and other complications, and increasing the risk of death for patients [24, 25]. Previous studies showed that polymorphisms of the *ALDH2* and *MTHFR* gene result in reduced enzyme activity may increase the risk of hypertension. However, the results of studies on the relationship between *ALDH2*, *MTHFR* gene polymorphisms and hypertension are inconsistent. In this study, *ALDH2* rs671 G/A, A/A genotypes and *MTHFR* rs1801133 C/T, T/T genotypes may be risk factors for hypertension in a Chinese Hakka population. This finding supports that *ALDH2* and *MTHFR* gene polymorphisms are associated with hypertension.

Mitochondrial ALDH2 is an enzyme responsible for metabolizing toxic aldehydes. Studies have shown that ALDH2 is a protective factor against oxidative stress, ALDH2 deficiency increases oxidative stress which is the predisposing factor of hypertension [13, 26]. There were some studies on the relationship between *ALDH2* gene polymorphisms and hypertension. Study has shown that *ALDH2* rs671 G/G genotype is a potent risk factor of hypertension among males in the general population in Japan [27]. *ALDH2* rs671 A/A genotype and A allele might increase the risk of hypertension, and *ALDH2* rs671 polymorphism might be a risk factor of hypertension in non-drinking Han Chinese [28]. On the contrary, there have also been some studies with opposite results. A study has shown that *ALDH2* rs671 A allele was a negative risk factor of essential hypertension in Mongolians from Inner Mongolia [29]. *ALDH2* rs671 G/A genotype and A allele were associated with a decreased risk of hypertension in drinkers, while drinkers carried A allele have lower SBP and TG level and higher HDL-C level [30]. People who carried the *ALDH2* rs671 A allele were less risk of developing hypertension, so *ALDH2* rs671 A allele is protective factor for hypertension in Han Chinese [31]. *ALDH2* rs671 A allele decreased risk of hypertension in men, but not women in a Chinese population in Zhejiang Province, China [32]. In addition, *ALDH2* rs671 polymorphism might be no correlated with hypertension in aged patients from Jiangsu Province, China [33]. In this study, *ALDH2* rs671 G/A, A/A genotypes may be risk factors for hypertension in a Chinese Hakka population.

MTHFR is one of the most important enzymes and plays a key role in the Hcy metabolism. Hyperhomocysteinemia is a risk factor associated with both hypertension and cardiovascular disease incidence. And the underlying mechanism is thought to be related to the functional disruption of vascular endothelial and smooth muscle cells [34, 35]. MTHFR is a key enzyme in the process of effecting the metabolism of Hcy. The level of MTHFR activity in vivo is closely related to the *MTHFR* gene polymorphisms. The *MTHFR* C677T polymorphism can reduce the activity of MTHFR and significantly alter the levels of a number of physiologically metabolites, including Hcy, folic acid, and vitamin [36]. Some studies have investigated the relationship between *MTHFR* gene polymorphisms and hypertension. Studies showed that there was no significant relationship between the *MTHFR* gene mutation and hypertension in Japanese [37, 38], Chinese [39], Danish [40], and Caucasian [41]. Conversely, some studies showed that *MTHFR* gene polymorphism was associated with hypertension. *MTHFR* rs1801133 T allele may contribute to hypertension in Argentineans from Buenos Aires city [42, 43]. *MTHFR* rs1801133 C/T genotype may be a risk factor of hypertension in a Caucasian population [44]. People carried *MTHFR* rs1801133 T allele enhanced the risk of hypertension among Chinese in Taiwan [45], Chinese Han population in Shihezi city, Xinjiang Province, China [46], Chinese from Jiangxi Province [47], and male Spaniard [48]. DBP and SBP levels of female hypertensive patients were different among the different genotypes of *MTHFR* in the Baiku Yao population in China [49]. In this study, *MTHFR* rs1801133 C/T, T/T genotypes may be risk factors for hypertension in a Chinese Hakka population.

According to the current data from this and other studies, the results on the relationship between *ALDH2* and *MTHFR* gene polymorphisms and hypertension are inconsistent. In related studies, the main reason for different or even opposite results is regional and ethnic differences. Mutations in the human *ALDH2* and *MTHFR* gene may be race-specific in a given region and region-specific in a given ethnic group [50]. In addition, it is also possible that these conflicting results may be due to their small sample sizes, or the patients’ existing conditions, such as occupation and daily exercise.

This observational study has some limitations. Firstly, this study is a single-center study and the sample size is not very large, which may lead to bias of the results. Secondly, this study is a retrospective study, there is maybe a certain selection bias because patients were selected from a medical institution. Thirdly, other genetic variations in *ALDH2* and *MTHFR* genes may influence the development of hypertension in this population. Future studies with larger sample sizes and more genetic variations of *ALDH2* and *MTHFR* genes are necessary to analyze this relationship.

## Conclusion

The present study showed *ALDH2* rs671 G/A, A/A genotypes and *MTHFR* rs1801133 C/T, T/T genotypes may be risk factors for hypertension in a Chinese Hakka population. This study contributes to the identification of people at high risk of hypertension, and facilitate the development of individualized strategies for the management of hypertension in the studied population. However, more researches are urgently needed to confirm this hypothesis in the future.

## Data Availability

The datasets used and analyzed during the current study available from the corresponding author on request.
